# Clinical outcomes of intramedullary tibial guides in total knee arthroplasty: experience from a single-centre cohort

**DOI:** 10.1186/s13018-025-06369-9

**Published:** 2025-10-22

**Authors:** Gennaro Pipino, Francesco Anzano, Enzo Claudio Moretti, Raffaele Borghi, Davide Corrado Vaccarisi, Patrizio Caldora, Bruno Violante, Nicola Maffulli, Filippo Migliorini, Vincenzo Salini

**Affiliations:** 1 Department of Orthopaedics, IRCCS Ospedale S. Raffaele, Milan, Italy; 2 Department of Orthopaedics, Villa Erbosa, Bologna, Italy; 3 Department of Orthopaedics, San Giuseppe Hospital, Gruppo Korian, Arezzo, Italy; 4 Department of Orthopaedics, Isola Tiberina Hospital, Rome, Italy; 5 Department of Trauma and Orthopaedic Surgery, Faculty of Medicine and Psychology, University La Sapienza, 00185 Rome, Italy; 6 School of Pharmacy and Bioengineering, Faculty of Medicine, Keele University, Stoke On Trent, ST4 7QB UK; 7 Centre for Sports and Exercise Medicine, Barts and the London School of Medicine and Dentistry, Mile End Hospital, Queen Mary University of London, London, E1 4DG UK; 8 Department of Trauma and Reconstructive Surgery, University Hospital of Halle, Martin-Luther University Halle-Wittenberg, Ernst-Grube-Street 40, 06097 Halle (Saale), Germany; 9 Department of Orthopaedic and Trauma Surgery, Academic Hospital of Bolzano (SABES-ASDAA), Via Lorenz Böhler 5, 39100 Bolzano, Italy; 10 Department of Life Sciences, Health, and Health Professions, Link Campus University, Via del Casale Di San Pio V, 00165 Rome, Italy

**Keywords:** Total knee replacement, Intramedullary guide, Extramedullary guide, Tibia

## Abstract

**Background:**

The use of an intramedullary (IM) or extramedullary (EM) tibial cutting guide in total knee arthroplasty (TKA) is debated. This study critically analyses our experience using the IM tibial cutting guide for TKA.

**Methods:**

This single-centre retrospective study included 369 patients who underwent TKA between January 1, 2023, and December 31, 2023. The data in the literature were analysed to evaluate the advantages and disadvantages of each of the two systems. The sample was radiographically assessed postoperatively to measure the medial proximal tibial angle (MPTA) and postoperative tibial slope (TS).

**Results:**

The mean MPTA was 91.6°, with 24% of patients achieving an alignment within an acceptable margin of error of ± 1°. The mean TS was 4.3°, with 45% of patients exhibiting a TS between 4° and 5°. The mean tourniquet time was 25 min, and 4 of 369 patients (1.08%) required blood unit transfusions. No tibial fractures or fat embolism were reported.

**Conclusion:**

Our study demonstrates satisfactory outcomes using the IM tibial cutting guide for TKA, with excellent alignment in both coronal and sagittal planes, short operative times, and low transfusion rates. The lack of consensus in the literature highlights the need for further research to draw definitive conclusions on the optimal tibial cutting guide technique for TKA.

**Level of evidence: III:**

## Background

Total knee arthroplasty (TKA) is the gold standard for end-stage osteoarthritis (OA), where severe pain and significant loss of range of motion (ROM) are predominant [1–4]. With consistently high patient satisfaction rates, TKA has established its role as a transformative procedure for individuals with severe knee OA [5–7]. TKA is among the most frequently performed orthopaedic procedures worldwide [8, 9].

The evolution of tibial cutting guides in TKA has shifted from intramedullary (IM) to extramedullary (EM) systems, spurred by concerns about accuracy and complications inherent to the IM approach. Initially, IM guides were preferred for their potential to align the tibial component with the tibia's anatomical axis of the tibia, thereby maintaining strictly mechanical alignment. However, they can also be used for other alignments. However, reliance on the intramedullary canal posed challenges, especially in patients with anatomical deformities such as tibial bowing or post-traumatic changes, leading to inaccuracies in component placement [10–12]. In a recent study, 72% of tibial components aligned correctly with IM guides, compared to 88% with EM guides [13]. Additionally, the risks of intramedullary rod instability and venous embolism have diminished the appeal of IM guides [11, 14]. In challenging cases, EM guides achieve comparable or superior alignment outcomes, prompting surgeons to increasingly prefer the EM technique [15, 16].

Despite the increasing global adoption of EM alignment, the debate between IM and EM guides remains, as both approaches present unique advantages and limitations [17–19]. For instance, IM guides produce fewer medial proximal tibial angle (MPTA) outliers compared to EM guides (9.5% vs 26.2%), with greater knee range of motion when alignment is within ± 3° of neutral [20]. The superior alignment of the IM technique could enhance long-term functional recovery [14]. Conversely, EM guides reduce postoperative bleeding and transfusion rates using less invasive techniques, underscoring the importance of patient-specific approaches [21].

This study aimed to assess our experience using the intramedullary tibial cutting guide for TKA. It evaluated the outcomes of all arthroplasty procedures performed in 2023 and compared them with existing literature.

## Methods

### Study design

This study was conducted in accordance with the Declaration of Helsinki, ensuring the ethical treatment of all participants involved. Written informed consent was obtained from each patient before their inclusion in the study, ensuring they understood the nature of the research, the procedures involved, and any potential risks associated with their participation. This research was designed and reported in accordance with the Strengthening the Reporting of Observational Studies in Epidemiology (STROBE) guidelines [22]. This study investigated the reasons for the observed shift in orthopaedic surgical practice from IM guide to EM guide. A thorough literature review identified the advantages and disadvantages of both techniques. The literature highlights five primary areas of concern that have garnered significant attention over the years: (1) the risk of adverse events associated with IM guidance, (2) blood loss during surgical procedures, (3) increased surgical times, and (4) alignment discrepancies in the coronal and sagittal planes. Each factor determines the most suitable tibial guide technique for orthopaedic surgeries. To better understand these dynamics, the present investigation analysed these parameters within our group of patients.

### Patient recruitment

For this retrospective single-centre single-surgeon cohort study, patients who underwent TKA between January 1, 2023, and December 31, 2023, at the III Orthopaedic Division of Villa Erbosa Hospital in Bologna, Italy, were considered. All patients treated during this period underwent TKA with intramedullary guidance. The inclusion criteria for the study comprised age between 30 and 90 years, an American Society of Anesthesiologists (ASA) score of ≤ 3, a Body Mass Index (BMI) ranging from 17 to 40, and a Hip-Knee Angle (HKA) between 18° of valgus and 18° of varus. Exclusion criteria were extra-articular deformities of the tibia or femur resulting from prior diaphyseal or metaphyseal fractures, fixation devices in the tibial canal, nickel-reported allergy, history of allergies to drugs used in perioperative and postoperative management and genetic mutations affecting coagulation cascade enzymes. Additionally, patients unable to provide autonomous informed consent from neurodegenerative disorders, learning disabilities, or psychiatric conditions were excluded.

### Surgical technique

**Fig. 1:**
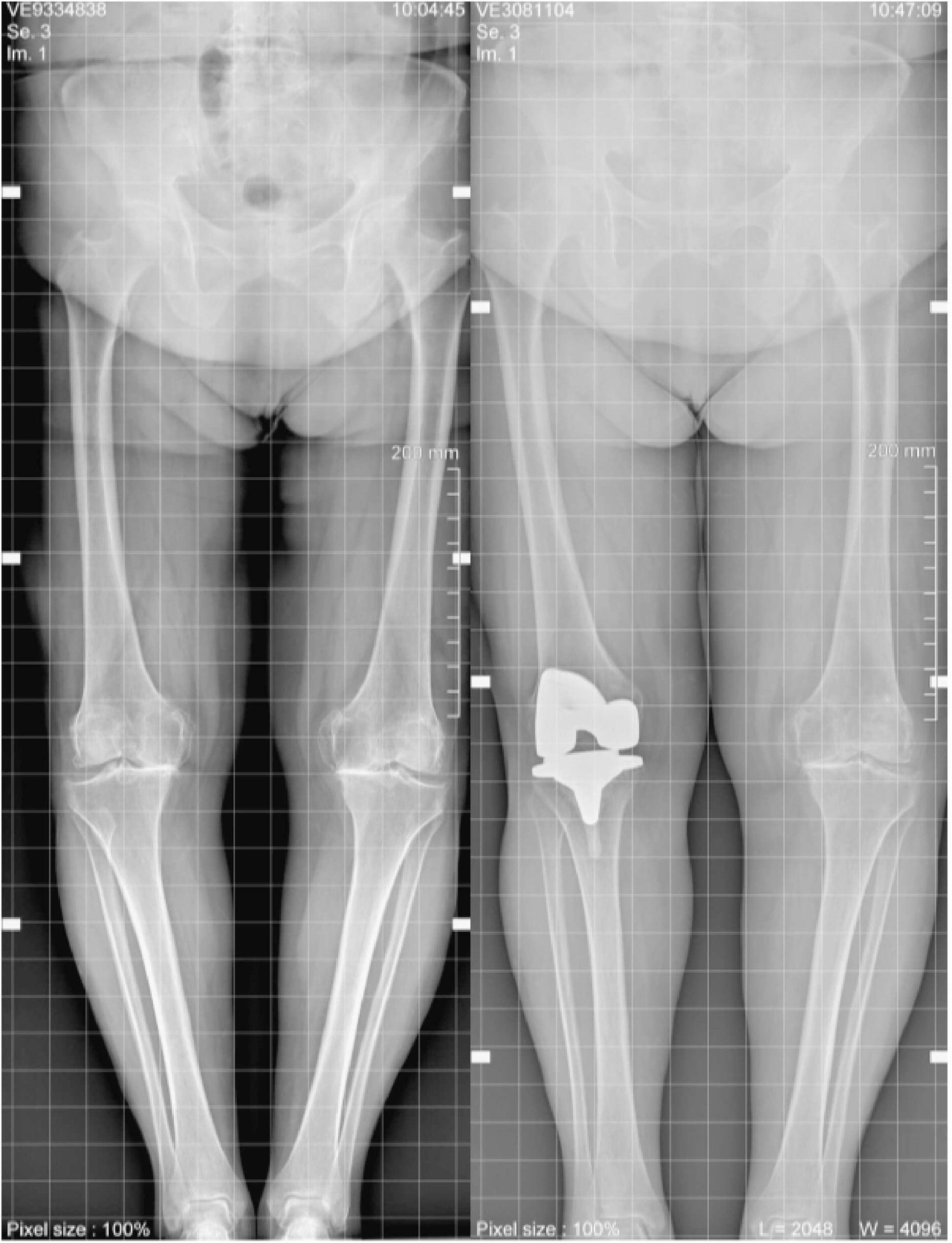
Radiographs of a female patient before surgery and 30 days postoperatively

All procedures were performed by the same senior surgeon, who has over 20 years of experience in TKA implantation. All patients received full-length standing radiographs the day before the operation and 30 days postoperatively at our hospital (Fig. 1). The radiographs were obtained in a true anteroposterior position, with no limb rotation, and the lateral radiograph of the knee was taken in a supine position with the knee at 30° of flexion. Only mechanical alignment (MA) was used to determine the correct axis of the cuts. All prosthetic models used for the implants were ATTUNE Knee System (Johnson & Johnson, New Jersey, US). Both the femoral and tibial components were cemented. Posterior stabilised inserts were used in all implants. The patella was not replaced in any patient. The surgical technique used in this study involved a median parapatellar incision, providing adequate exposure while minimising disruption to surrounding soft tissues. The 4000 TS Tourniquet System (Zimmer Biomet, Warsaw, USA) is positioned before the sterile field is established. It is inflated before the incision and deflated immediately after the implantation of the definitive components. A minimal medial parapatellar approach was employed, particularly concerning the vastus medialis. For suturing, Stratafix absorbable double needles (Johnson & Johnson, New Jersey, US) were used to close the capsular layer. Monocryl (Johnson & Johnson, New Jersey, US) was used to close the subcutaneous tissue. Finally, metal staples were used to suture the skin. A vial of tranexamic acid 500 mg/5 ml is used in venous infusion during the operation, and two vials are used instead at a subfascial level, injected with a syringe after suturing the fascia. Intra-articular drainage was applied in all patients and removed on the first postoperative day. From postoperative day one, all patients were encouraged to walk with crutches weight-bearing and received physical therapy in the subsequent days. The mean time to discharge was five days.

### Data of interest

The patients' demographic data were collected, and the body mass index (BMI) was calculated for each patient. All radiographic assessments were performed independently by two assessors who were not directly involved in the patients' clinical management. Each assessor took the measurements three times, and the mean value obtained was used for subsequent analysis. Radiographic evaluations measured each patient's medial proximal tibial angle (MPTA) and postoperative tibial slope (TS). Radiographic measurements were obtained using Intellispace PACS (Philips, Amsterdam, the Netherlands) software at 1-month follow-up on full-length weight-bearing radiographs. TS measurements were calculated on sagittal radiographs, using the tibial anatomical-mechanical axis as a reference. The perpendicular was drawn, and the angle formed between the perpendicular and the straight line passing through the highest anterior and posterior extreme points of the tibial plateau was measured. The MPTA measurements were calculated by taking the tibial anatomical-mechanical axis and its perpendicular as a reference; the angle formed between the line parallel to the bone resection and the perpendicular to the axis was then evaluated.

Additionally, the patient's preoperative and postoperative haemoglobin (Hb) levels were assessed, considering the number of transfusions administered across the total number of patients operated on. For the patients pre-admission timelines, preoperative Hb values were evaluated the day before the surgery, while postoperative Hb values related to the first postoperative day. Furthermore, the duration of the tourniquet use was recorded for each surgical procedure.

### Statistical analysis

All statistical analyses were conducted by the primary investigator using the IBM SPSS software version 25 (Armonk, US). Continuous variables were analysed using mean differences, with a 95% confidence interval (CI).

## Results

### Patient enrolment

**Fig. 2:**
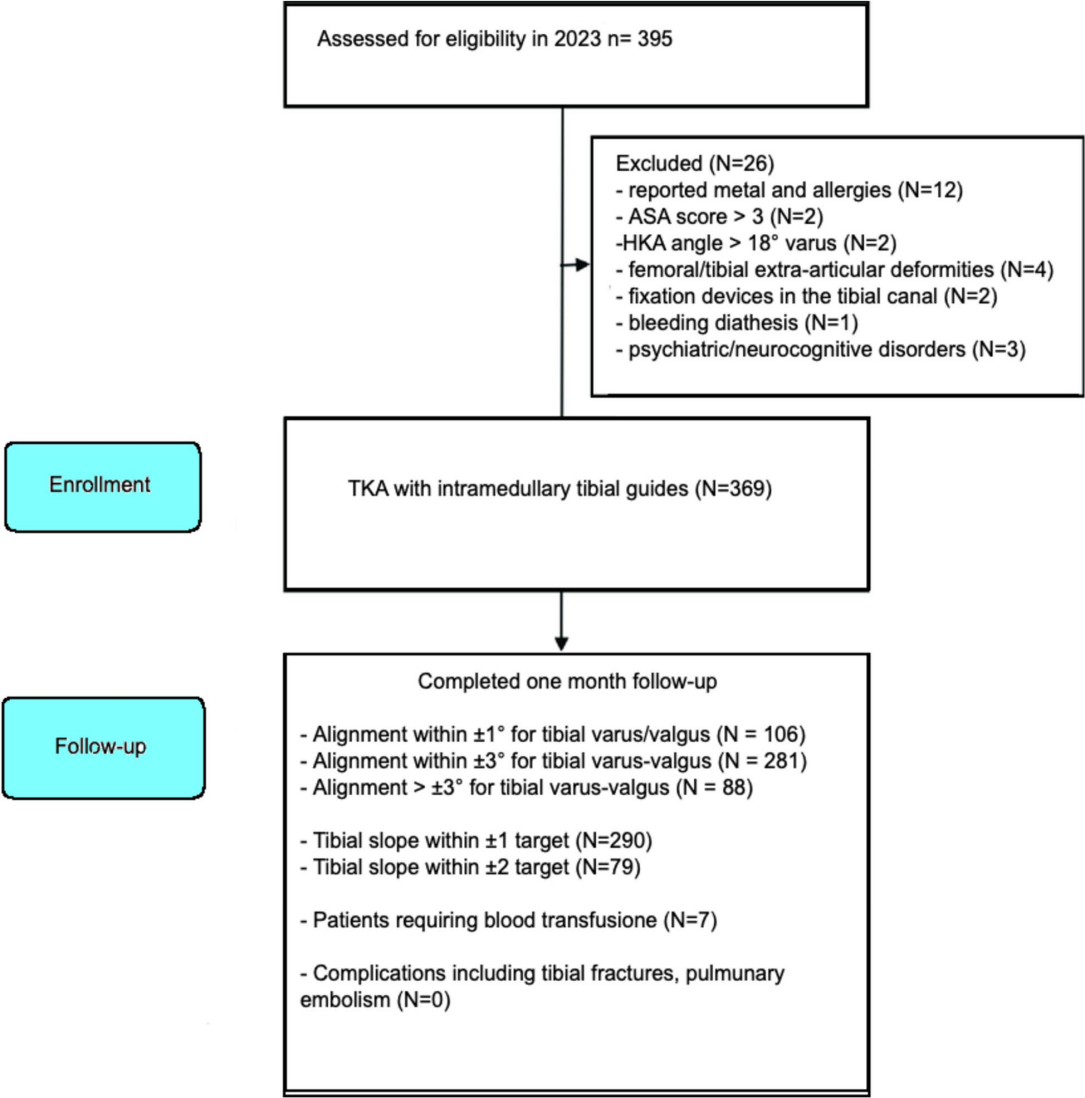
STROBE flowchart of the patient enrollment

During the whole of 2023, a total of 395 patients underwent TKA at the III Orthopaedic Division of Villa Erbosa Hospital in Bologna, Italy. Of these, 26 patients were excluded from this study as they did not match the eligibility criteria: reported metal allergy (N = 6), drug allergies (N = 6), ASA score > 3 (N = 2), HKA angle > 18° varus (N = 2), femoral extra-articular deformities (N = 1), tibial extra-articular deformities (N = 3), presence of fixation devices in the tibial canal (N = 2), genetic mutations affecting coagulation cascade enzymes (N = 1), psychiatric condition (N = 1), and neurodegenerative disorders (N = 2). Patients with severe (> 18°) and extraarticular deformities were treated with implants with higher constraints, with tools using EM guides. Patients who reported metal allergies were treated with a different non-allergic implant using EM guides. A total of 369 patients meeting the eligibility criteria were enrolled in the study (Fig. 2).

### Patient demographic

A total of 369 patients who underwent TKA at the III Orthopaedic Division of Villa Erbosa Hospital in 2023 were enrolled for this study (158 men and 211 women). The average age of the patients was 62 ± 10.1 years (36–84). The mean body mass index (BMI) was 29.7 kg/m^2^ (range, 21.3–39.2 kg/m^2^). Generalities of the patients are reported in Table 1.

**Table 1:** Demographics of the population (N = 369) who underwent TKA with IM guides

Number of patients	369
Age at surgery (*years*)	62 ± 10.1 (36–84)
Gender (*female/male*)	211 (57.2%) / 158 (42.8%)
BMI (*kg/m*^*2*^)	29.7 ± 3.8 (21.3–39.2)
Mean MPTA (°)	91.6 (84.5 varus to 93.7 valgus)
Mean tibial slope (°)	4.3° (1.2–6.2)
Mean tourniquet time (*minutes*)	32 ± 5 (22–42)
Mean preoperative Hb level (*mg/dL*)	13.7 (8.8–17.6)
Postoperative mean HB levels (*mg/dL*)	11.7 (14.6–7.7)

Continuous variables are expressed with the main values, standard deviation and range of values (in parentheses). Absolute values are represented by frequencies and relative percentages (in parentheses)

### Imaging measurements

Radiographic measurements evaluated the accuracy of pre- and intraoperative surgical planning. The mean MPTA was 91.6° (range 84.5° varus to 93.7° valgus). The distribution of MPTA values revealed precise surgical alignment: in 106 prostheses, the MPTA was 89° to 91°; in 78, 87° to 89°; in 82, 84.5° to 87°; in 97, 91° to 93°; and in six prostheses, it was 93° to 93.7°. TS measurements, targeting a range of 3°–5°, yielded a mean value of 4.3° (range, 1.2°–6.2°). 125 patients presented a TS between 3°–4°, and 165 patients had a TS between 4°–5°. 53 patients had a TS below 3°, and in 26 patients the TS exceeded 5°.

### Surgical time

The mean tourniquet time was 32 ± 5 min, ranging from 22 to 42 min.

### Blood loss

The average preoperative Hb-level among the 369 patients included in this study was 13.7 mg/dL, with a maximum value of 17.6 mg/dL and a minimum of 8.8 mg/dL. Postoperative mean haemoglobin levels were 11.7 mg/dL (14.6 mg/dL to 7.7 mg/dL). 7 of 369 (1.89%) patients required blood transfusions for postoperative anaemia. The mean difference between preoperative and postoperative Hb levels was 2.07 mg/dL (4.4 mg/dL to 0.4 mg/dL).

### Complications

No patients experienced fat embolism or tibial fractures.

## Discussion

According to the main findings of the present study, 28.8% (N = 106) of patients achieved an alignment, measured by the MPTA, that fell within a narrow margin of error of ± 1° for varus/valgus, considering the strictly mechanical alignment desired. 76.2% (N = 281) of the patients remained within an error range of ± 3° for tibial varus-valgus. Only 23.8% (N = 88) of the patients experienced a more borderline resection; 22.2% (N = 82) showed a tendency toward varus alignment. The findings of the present study align with previous randomised and meta-analytic evidence reporting comparable outcomes between intramedullary and extramedullary tibial guides in TKA. Nevertheless, our results contribute complementary real-world data, showing that intramedullary guides can provide accurate alignment and acceptable tibial slope, with low transfusion rates and no observed complications, in a large consecutive cohort. Although the study design and sample size cannot capture rare adverse events, the consistency of these outcomes supports the view that, when applied with careful patient selection and surgical expertise, intramedullary guides remain a safe and effective option in contemporary TKA practice [16, 20, 23–25]. Currently, there is no significant difference in alignment outcomes between IM and EM guides. Some studies [26–31] argue that alignment outcomes between the two techniques are comparable. A meta-analysis [32] of 1000 patients revealed no significant postoperative radiographic differences between IM and EM guide systems. El Nahas et al. [33] divided 100 patients, all operated by the same surgeon, into two groups: one receiving IM guidance and the other EM guidance. The target MPTA of 90° was achieved in 22% of the patients in the IM group compared to 13% in the EM group [33]. The literature is replete with conflicting evidence on this topic. More outlier patients, relative to the desired tibial cut, are observed using EM guides [34]. In contrast, Chin et al. [35] and Mizuuchi et al. [36] report more outliers when using IM guides [34, 37]. This divergence highlights the ongoing debate within the orthopaedic community regarding the comparative effectiveness of IM and EM guides in achieving optimal tibial coronal alignment.

Nevertheless, despite the discrepancy in the alignment values between IM and EM, the satisfaction indices, such as the Knee Society Score (KSS), Functional Knee Society Score (FKSS), and Visual Analogue Score (VAS), remain comparable, and the ROM does not undergo significant variations [20].

Indeed, the second metatarsal is not an accurate landmark for correct alignment in the coronal plane [38]. In addition, the centre of the ankle does not precisely correspond to the midpoint between the malleoli, being slightly medial to this point (5–10 mm). Furthermore, in obese patients, it may be difficult to identify [39]. Bypassing anatomical reference points in obese patients with an intramedullary cutting guide reduces operative time [40], without changes in alignment. No changes in alignment were reported, also in the non-obese population [27]. The population in the present investigation was not selected based on their BMI (we, however, use a BMI of 40 as a limit for offering surgery), nor for age and ASA score, making our results more generalizable.

Regarding tibial alignment in the sagittal plane, the TS was evaluated. 45% of patients in whom IM alignment guides were used exhibited a TS between 4° and 5°, and 34% of patients achieved the target intraoperative range of 3°–4°, while 23% were outliers. Most outliers had a lower posterior slope, with TS values < 3°. IM guides could potentially allow for a more spatially accurate positioning near the posterior margin of the tibia, which, in theory, might enable a more precise estimation of the tibial slope. The scientific literature on this topic is poor, with limited sources comparing TS outcomes according to IM and EM alignment guides [32]. In larger cohorts, no statistically significant differences in TS between the two systems were found [31]. The limited literature favours EM guides to achieve a more accurate TS by replicating the patient's preoperative TS more than IM guides [41, 42]. Similarly, IM guides were associated with a higher margin of error in underestimating the TS intraoperatively, which led to augmented radiolographic values (4° IM vs. 2.7° EM). This trend, supported by other investigations [41, 42], contrasts sharply with the conclusions of this work, which, as in the study by Chiu et al. [43], found a tendency to lower TS with the IM guide. In the present cohort, most outliers fell below the 3° TS threshold, indicating that IM guides tended to produce a reduced TS with less posterior inclination. The experience of the operating surgeon likely influences this discrepancy in results. All TKA procedures in the present work were performed by a single experienced surgeon accustomed to using IM guides. Notably, these results were observed despite using traditional cutting techniques, without intraoperative navigation or robot-assisted systems, suggesting that the use of an IM guide may have contributed to this outcome. Based on recent studies worldwide, approximately one-third of TKA procedures are performed with robotic-assisted techniques [44, 45]. This implies that approximately 66% of TKAs use traditional methods. The mean tourniquet time was 32 min, a relatively short duration. This represents a clear advantage, considering the reduced risk of infection associated with shorter surgical times [46, 47]. Comparative analyses have reported shorter tourniquet time using IM guides compared to EM guides [31, 40]. Maestro et al. [25] found a not statistically significant difference of 10 min in tourniquet time between the two techniques. Similarly, Bian et al. [23] reported mean tourniquet time values of 79 min for the IM and 84 min for the EM techniques. The tourniquet time observed in the present investigation may also reflect the surgeon's extensive experience with this procedure.

Only 4 of our 369 patients (1.08%) required blood unit transfusions, with the indication for transfusion strictly adhering to protocol-defined thresholds of < 8 mg/dL Hb. The mean Hb loss of approximately 2 mg/dL is likely attributable to meticulous intraoperative haemostasis and reduced overall surgical time. Although the literature offers limited evidence on blood loss comparisons between the two, most studies suggest a reduction in haemoglobin loss associated with EM guides [25, 48]. Conversely, a large-scale survey involving 883 patients found no significant differences in transfusion rates or haemoglobin drop between IM or EM guides [49]. On the other hand, there is substantial evidence linking blood loss to femoral canal violation. Several studies have observed reduced transfusion rates and lower blood loss in patients treated with extramedullary alignment systems for femoral resection guides compared to those using intramedullary systems [50–52]. Femoral factors, rather than tibial ones, may play a greater role in postoperative bleeding, thereby challenging the notion that tibial EM guides inherently reduce postoperative anaemia.

In the present study, no tibial fractures or fat embolism were observed. However, the literature occasionally associates these complications with the use of IM guides, and these are among the main reasons that have prompted orthopaedic surgeons to stop using IM guides. For instance, a higher rate of fat embolism was documented in patients treated with IM guides [53]. Supporting this, a recent echocardiography imaging study evidenced more emboli in the right atrium in patients undergoing IM TKA guides compared to those treated with EM [48]. Notably, these findings remained purely radiological and did not translate into a higher rate of adverse clinical events for IM guides. Similarly, another study reported higher rates of these postoperative complications in the IM group but deemed the results not statistically significant [23]. Conversely, another study [54] noted not only an increased risk of fat embolism with IM guides but also a higher incidence of tibial fractures. In contrast, O'Connor suggested no significant difference in fat embolism rates between IM and EM guides [55]. One additional study highlighted the lack of definitive evidence linking IM guides to a higher risk of tibial fractures. However, in patients with severe diaphyseal deformity, IM guides could theoretically increase this risk [27]. Interestingly, the same study also refuted the elevated risk of fat embolism associated with IM guides, further contributing to the nuanced understanding of these potential complications.

This study has several limitations. The retrospective design and the single-centre, single-surgeon setting reduce the external validity of the findings, although this homogeneity ensured consistency in the surgical technique and perioperative management. The lack of a direct comparative group treated with extramedullary guidance prevents head-to-head conclusions between the two systems. Although the cohort was relatively large, it remains insufficient to draw reliable conclusions on rare but clinically relevant complications such as fat embolism or tibial fractures. In addition, no gender or previous activity level differences were considered, nor were clinical and functional scores reported, which limits the ability to correlate radiographic accuracy with functional recovery and patient-reported outcomes. The study also did not assess coronal alignment, which might have further validated the reproducibility of radiographic measurements. Finally, the study focused exclusively on conventional instrumentation; robotic and navigation-assisted systems are used in clinical practice. Functional alignment techniques have recently emerged as an alternative approach that avoids violation of both the tibial and femoral medullary canals, aiming to restore patient-specific kinematics while minimising invasiveness. We are conducting a randomised controlled trial comparing robotic-assisted functional alignment with freehand TKA to evaluate potential differences in alignment accuracy and clinical outcomes [56, 57]. Although these limitations may limit the perceived novelty of our work, it is important to emphasise that conventional TKA still represents the majority of procedures performed worldwide. Therefore, real-world evidence on intramedullary guides retains clinical value.

## Conclusions

The results demonstrate low required transfusions, excellent alignment in both the coronal and sagittal planes and short operative times. The lack of consensus highlights the complexity surrounding tibial intramedullary guides in total knee arthroplasty. At the same time, discrepancies in findings emphasise the need for further research and more robust datasets to reach definitive conclusions.

## Data Availability

The datasets generated during and/or analysed during the current study are available throughout the manuscript.
